# 

**Published:** 1887-11-12

**Authors:** 


CONTENTS.

**A Gentleman with a Grievance** - - - - - 107

**Words of Consolation.**—Chapter LVIII. The Sufferings of Christ - - - - - 108

**The Doctor's Pulpit.**—Woman at Home - - - - 109

**The Common Accidents of Every Day Life.** By R. A. P. FORRESTER, B.A. - - - - - 110

**Medicine Men of the World.** By the MAN IN THE CROWD. —III. Medicine Men in Savage Lands - - - 111

**The National Pension Fund** - - - - - 112

**The Sick, the Churches, and the Hospitals** - - 112

**Matrons' Corner** - - - - - 112

**Professional Notes.** By GEO. W. POTTER, M.D. - - 113

**The Printing Trade and the Hospitals** - - - 114

**Notes and News.**—Hospitals in 1698—1703.—Nurses' Homes.—Fifth of November Accidents.—Vacancies.—Mirth and Madness.—Frederick Street Industrial School and the Consumption Hospital, Belfast.—Hospital Sunday Collection at Birmingham.—Worth Making a Note of!—East Suffolk and Ipswich Hospital and its Medical Officers.—Canon Machell on the Hospital Sunday Fund.—The Judge and the Hospital.—Collections in London on Hospital Sunday, &c., &c. - - - 114

**Annotations.**—A Dangerous Climate.—Underclothing.—Neighbours or Strangers? - - - - - 117

**Amusements with Profit, for all Ages.**—For Men and Women.—The Theatres.—The Children's Corner - - 118

**East and West.** By LESLIE KEITH. Chapter VII. An Explorer 119

**On Hospital Construction.** By KEITH D. YOUNG, F.R.I.B.A. 121

**Hospitals, Doctors, Treatment, and Attendance.** The In- and Out-Patients' Hospital Diary - - - 123

**The Students' Column** - - - - - 124

